# Performance and characterization of phenol-formaldehyde resin with crude bio-oil by model compound method

**DOI:** 10.1371/journal.pone.0271478

**Published:** 2023-01-10

**Authors:** Yuxiang Yu, Xiaoqian Qiu, Chao Li, Defu Bao, Jianmin Chang

**Affiliations:** 1 College of Art and Design, Zhejiang Sci-Tech University, Hangzhou, Zhejiang, China; 2 Lab of Material Innovation Design and Intelligent Interaction, Zhejiang Sci-Tech University, Hangzhou, Zhejiang, China; 3 College of Materials Science and Technology, Beijing Forestry University, Beijing, China; University of Pisa Department of Biology: Universita degli Studi di Pisa Dipartimento di Biologia, ITALY

## Abstract

In order to clarify the effects of crude bio-oil for phenol-formaldehyde resin, the phenol-formaldehyde resin with bio-oil model compounds (BMPF) were prepared by model compound method. The bonding strength and aging resistance of BMPF were determined, and their microstructure and chemical bonds were also analyzed by scanning electron microscope, Fourier transform infrared spectroscopy, and nuclear magnetic resonance analysis, respectively. The results showed that the components of crude bio-oil had various degrees of effects on the BMPF performance, and the most obvious one is the phenols. The phenols and the ketones of bio-oil had positive effects on the bonding strength. The ketones had the biggest effect on the surface smoothness of BMPF film. But all components of bio-oil could inordinately improve the aging resistance of BMPF. The structural analysis indicated that the effects of bio-oil components on the BMPF performance by changing the resin structure. The CH_2_ peak in FT-IR and the methylene bridges intensity in NMR of phenol-free BMPF and ketone-free BMPF were smaller, while the results of aldehyde-free BMPF and acid-free BMPF were opposite. And the influence degree of BMPF structure was basically consistent with that of BMPF performance. These results could provide a basis for the modification of phenol-formaldehyde resin by crude bio-oil.

## Introduction

Phenol-formaldehyde resin (PF) is widely used in wood-based panel because of its excellent mechanical properties, thermal stability, flame retardancy, and water and chemical resistance [1, 2]. Phenol, the main raw material in PF, is produced from petroleum resources. However, petroleum is a non-renewable natural resource and its continued use is not sustainable. Therefore, in recent decades, many researchers are shifting focus towards renewable materials as a substitute for phenol to produce bio-based PF, such as lignin [3, 4], tannin [5], and cardanol [6]. Compare with PF, lignin-based PF showed higher adhesion strength [4], tannin-based PF had less impact on the environment [5], and cardanol-based PF had better thermal performance [6].

Bio-oil, the main product from the rapid pyrolysis of biomass, has been successfully used in the preparation of PF in recent years [7–9]. Compared with the petroleum-based PF, the bio-oil phenol-formaldehyde resin (BPF) has similar chemical and physical properties, but a better price competitiveness [10–15]. However, the components of bio-oil are complex, including hundreds of organic components such as phenols, ketones, aldehydes, acids and sugars [7, 16–18]. Some organic components in the bio-oil can directly take part in the resin reaction, resulting in the change of resin structure and properties. The other components can also affect the resin properties as fillers. In order to enhance the beneficial effects of bio-oil on the resin synthesis, it is of great significance to clarify the effects of different components in the bio-oil.

Model compound method is a common way to use artificial compounds with same or similar structure and function to simplify the complex compound, such as bio-oil. Liu et al. [19] selected the blend of acetic acid and acetone as bio-oil model compound to evaluate the catalytic performance of 3D-LaNiO_3_ perovskite for H_2_ production and found that the 3D-LaNiO_3_ catalyst showed better catalytic performance. Fortunate et al. [20] used 2-hydroxybenzaldehyde as bio-oil model compound to delineate the effect of process variables on the catalytic hydrodeoxygenation of bio-oil. Tran et al. [21] studied the influence of Fe/AC and Ni/gamma-Al_2_O_3_ catalysts on the hydrodeoxygenation of woody bio-oil using guaiacol as model compound. Wang et al. [22] clarified the aging mechanism of bio-oil by evaluating the aging performance of 39 kinds of bio-oil model compounds, and found that the acids played an important role during aging.

In this study, the crude bio-oil was simplified into five groups, including phenols, ketones, aldehydes, acids and sugars, according to its component distribution. And the bio-oil and its component model compounds were formulated by model compound method. The phenol-formaldehyde resin with bio-oil model compounds (BMPF) were prepared, and their bonding strength and aging performance were determined. The microstructure and chemical bonds of BMPF were also analyzed to clarify the effects of crude bio-oil for PF.

## Materials and methods

### Materials

Bio-oil, an acid liquid (pH 3.5), was obtained by the fast pyrolysis of *Larix gmelinii (Rupr*.*) Kuzen* in a fluidized bed at 550°C for 2–3 s by the Lab of Fast Pyrolysis of Biomass and Productive Utilization (Beijing Forestry University, Beijing, China). And the organic components of bio-oil were displayed in Table 1 by gas chromatographic-mass spectrometric (GC-MS) analysis. Phenol, formaldehyde (aqueous solution, 37 wt.%), sodium hydroxide (NaOH), guaiacol, catechol butanone, cyclopentane dione, vanillin, furfural, D-glucose, acetic acid, phenylacetic acid were supplied by Xilong Chemical Industry, Guangdong, China. Poplar veneers (8% moisture content, 400 mm × 400 mm × 1.5 mm) were provided by Xinda wooden Co., Ltd., Hebei, China.

**Table 1:** GC-MS of bio-oil.

Compounds	Molecular Formula	Peak Area (%)
**Phenols**		**33.42**
Guaiacol	C_7_H_8_O_2_	6.52
4-methylguaiacol	C_8_H_10_O_2_	5.72
Phenol	C_6_H_6_O	3.16
4-methylcatechol	C_7_H_8_O_2_	3.65
Catechol	C_6_H_6_O_2_	2.64
P-methylphenol	C_7_H_8_O	1.44
2-methoxy-4-propylphenol	C_10_H_14_O_2_	1.39
Isoeugenol	C_10_H_12_O_2_	1.39
2-cresols	C_7_H_8_O	1.20
4-ethylguaiacol	C_9_H_12_O_2_	1.04
4-ethylresorcinol	C_8_H_10_O_2_	0.90
3,4-xylenol	C_10_H_12_O_2_	0.88
4-methyl-2- methoxy-6 propenyl phenol	C_10_H_10_O_3_	0.82
Eugenol	C_10_H_12_O_2_	0.74
4-Ethylguaiacol	C_9_H_12_O_2_	0.69
2-methyl-1,4catechol	C_7_H_8_O_2_	0.52
2,6-dimethylphenol	C_8_H_10_O	0.39
Trimethylhydroquinone	C_9_H_12_O_2_	0.33
**Ketones**		**29.56**
2-Butanone	C_4_H_8_O	6.25
3-Methyl-1,2-cyclopentanedione	C_6_H_8_O_2_	5.89
4,6-dimethyl-2-pyrone	C_7_H_8_O_2_	4.22
4-methyl-2(5H)-furanone	C_5_H_6_O_2_	3.81
2-propyl-2-ethylcyclohexanone	C_11_H_20_O	3.30
5-methyl-2-furanone	C_5_H_6_O_2_	2.70
Acetovanillone	C_9_H_10_O_3_	1.54
3-ethyl-2-hydroxy-2-cyclopenten-1-one	C_7_H_10_O_2_	1.36
2-hydroxy-6-methoxyacetophenone	C_9_H_10_O_3_	0.49
**Aldehydes**		**13.45**
Vanillin	C_8_H_8_O_3_	7.03
Furfural	C_5_H_4_O_2_	2.87
5-acetonyl-2-furaldehyde	C_8_H_8_O_4_	2.34
Butanal	C_4_H_8_O	1.21
**Acids**		**9.33**
Acetic Acid	C_2_H_4_O_2_	7.13
4-hydroxy-3-methoxyphenylacetic acid	C_9_H_10_O_4_	1.29
2,2-dimethylglutaric acid	C_7_H_12_O_4_	0.75
Camphoric acid	C_10_H_16_O_4_	0.16
**Sugars**		**10.05**
D-mannose	C_6_H_12_O_6_	9.26
*β*-D-allose	C_16_H_12_O_6_	0.79
**Others**		**4.19**
2-ethyne	C_6_H_10_	1.43
1,9-decadiene	C_10_H_18_	1.35
2,4-diacetoxypentanel	C_9_H_16_O_4_	0.27
Hepty lacetat	C_8_H_10_O_5_	0.37
2,5-dimethyl-3-hexanol	C_8_H_12_O	0.34
N-methylpiperidine	C_6_H_13_N	0.43

### Preparation

According to the results of GC-MS analysis of bio-oil (Table 1), the model compounds of bio-oil were configured according to the corresponding proportions (Table 2). The formulas of bio-oil model compounds were detailed in Table 3.

**Table 2:** Composition and ratio of bio-oil model compounds.

Model compound	Composition	Ratio
Phenols	Phenol; guaiacol; catechol	4:1:2
Ketones	Butanone; cyclopentane dione	1:1
Aldehydes	Vanillin; furfural	7:3
Sugars	D-glucose	——
Acids	Acetic acid; phenylacetic acid	5:3

**Table 3:** Preparation of bio-oil model compounds.

Model compounds	Phenols	Ketones	Aldehydes	Acids	Sugars	Water
Bio-oil model	23%	21%	9%	6%	7%	30%
Phenol-free bio-oil model	——	21%	9%	6%	7%	30%
Ketone-free bio-oil model	23%	——	9%	6%	7%	30%
Aldehyde-free bio-oil model	23%	21%	——	6%	7%	30%
Acid-free bio-oil model	23%	21%	9%	——	7%	30%

The BMPFs were synthesized according to Yu [23]. The molar ratio of phenol (including bio-oil model compounds) to formaldehyde was 1:2. The substitute rate of bio-oil to phenol was 20 wt.%, and the addition amount of NaOH was 20 wt.% of the mass of phenol. The BMPFs were denoted as BMPF, phenol-free BMPF, ketone-free BMPF, aldehyde-free BMPF and acid-free BMPF. And the BPF was prepared as the control group.

The BMPF plywood and films were prepared according to Yu [24]. After keeping for 48 h in the room, the plywood was sawed into samples with dimension of 100 mm × 25 mm × 3 mm, and 5.3 ± 0.3 g samples were selected as plywood samples. The cured resin films (80 mm × 10 mm × 2 mm) were kept in temperature humidity chamber at 20 ± 2°C and 65 ± 5% relative humidity for 48 h. The sample with a mass of 1.7 ± 0.2 g was selected as the resin film samples.

### Analysis

The plywood and resin film samples were examined by a UV accelerated weathering tester (Yiheng Co., Ltd., Shanghai, China) according to the ASTM G 154. Each 12 h weathering cycle consisted of 8 h of UV exposure at 60°C and 4 h condensation at 50°C. The sample number of plywood and resin films was 6 and 3, respectively. The GC-MS analysis of bio-oil was recorded on a GC/MS-QP system (Shimadzu, Kyoto, Japan) with 20°C/min heating rate. An inlet temperature of 250°C, a He gas injection volume of 0.5 μL and a flow split-ratio of 50:1 were used for the GC. A junction temperature of 260°C, an EI source electron energy of 70 eV and a scan range of 29–500 amu were used for MS.

The moisture content of bio-oil was calculated by V30 Karl-Fischer moisture meter (Mettler Toledo, Zurich, Switzerland). The pH, viscosity, and solid content of resins were tested by China National Standards GB/T 14074–2017. Each experiment was conducted at least three times.

The formaldehyde emission and bonding strength of plywood was evaluated on the basis of China National Standard GB/T 17657–2013. The formaldehyde emission was detected by the dryer method. After being placed at relative humidity of 65±5% and 20±2°C for 7 days, the plywood was put into the dryer of crystallization dish with distilled water and heated in a constant temperature and humidity box. Measure formaldehyde emission by sucking distilled water from crystallization dish. The experiment of formaldehyde emission was conducted three times, and the sample number of bonding strength test was 6.

The scanning electron microscope (SEM) analysis of resin films were measured by SU8010 SEM (Hitachi, Tokyo, Japan) at 5.0  kV accelerating voltage. The Fourier transform infrared spectroscopy (FT-IR) analysis of resins were tested by a Nicolet iS5 FT-IR (Nicolet, Wisconsin, USA) over the range of 400 to 4000 cm^−1^ with a 4 cm^−1^ resolution and 64 scans. The solid state nuclear magnetic resonance (NMR) analysis of resins was acquired at a frequency 100 MHz using JNM-ECZ600R (JEOL, Tokyo, Japan).

## Results and discussion

### FT-IR analysis

**Fig 1:**
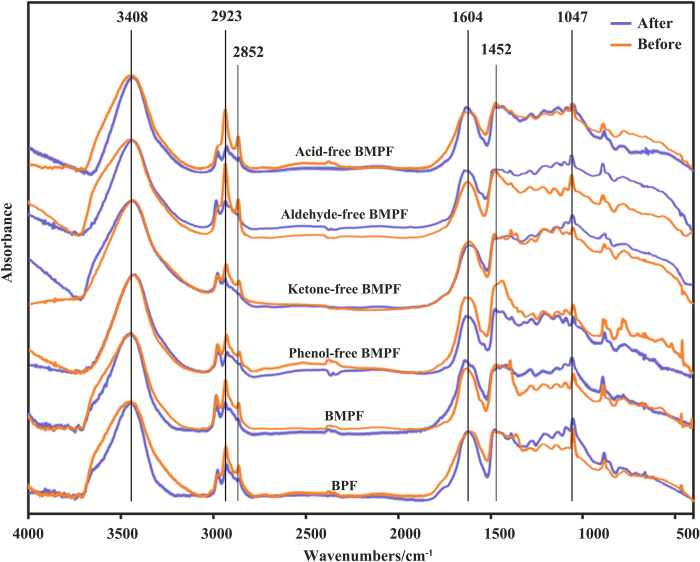
FTIR curves of BMPFs before and after aging for 960 h.

The FT-IR curves of BMPFs before and after aging for 960 h are displayed in Fig 1, and the attribution of characteristic absorption peaks is shown in Table 4 [10, 13, 14, 23, 24]. As shown in Fig 1, the peak of methylene (CH_2_) groups at the region of 2924 cm^−1^ and 2854 cm^−1^, which can indirectly represent the polymerization degree of PF resin [25, 26]. The CH_2_ groups peak of BMPF was stronger than that of BPF, because the phenolic substance activity of bio-oil model compound was stronger than that of bio-oil. Compared with BMPF, the CH_2_ groups peak of phenol-free BMPF was weaker, because the CH_2_ groups were generated by the reaction of phenol and aldehyde, and the lack of the phenols reduced the CH_2_ content in the resin. The weakening of CH_2_ groups peak of ketone-free BMPF also confirmed the promoting effect of the ketones on resin polymerization. The increase of CH_2_ groups peak of aldehyde-free BMPF and acid-free BMPF meant that the aldehydes and the acids in the bio-oil have a negative effect on the resin synthesis. The peak of ether bond (C—O—C) groups at the region of 2924 cm^−1^ and 2854 cm^−1^. Ether bond is another connecting structure between molecular chains of PF resin, which can also indirectly represent the degree of resin polymerization. Compared with BPF, the changes on the ether bond peak of BMPFs were similar to those of CH_2_ peak.

**Table 4:** Peaks and assignment of FT-IR spectra for BMPFs.

Wave number (cm^-1^)	Vibration	Assignment
3408	ν(—OH) ^a^	Phenolic OH and aliphatic OH stretching vibration
2923, 2852	ν(CH_2_)	Aliphatic CH_2_ asymmetric stretching vibration
1604, 1452	ν(C = C)	C = C aromatic ring stretching vibration
1047	ν(C—O—C)	Phenolic C—O—C stretching vibration

^a^ ν: Stretching vibration.

After aging for 960 h, the peak of CH_2_ and C—O—C groups decreased significantly, indicating that the polymerization degree of resin decreased after aging. Compared with BPF, the CH_2_ peak of BMPF decreased significantly. It showed that other substances in the bio-oil could reduce the polymerization degree, but improved the aging property of resin. Compared with BMPF, the CH_2_ groups peak of phenol-free BMPF decreased significantly after aging, which might due to that resin failed to form a good network structure because of the lack of phenols. The CH_2_ groups peak of ketone-free BMPF weakened the biggest. It might be due to the lack of ketones, which increased the contact area of external environment (water, light and other aging factors), and aggravated the aging degree [27].

### NMR analysis

**Fig 2:**
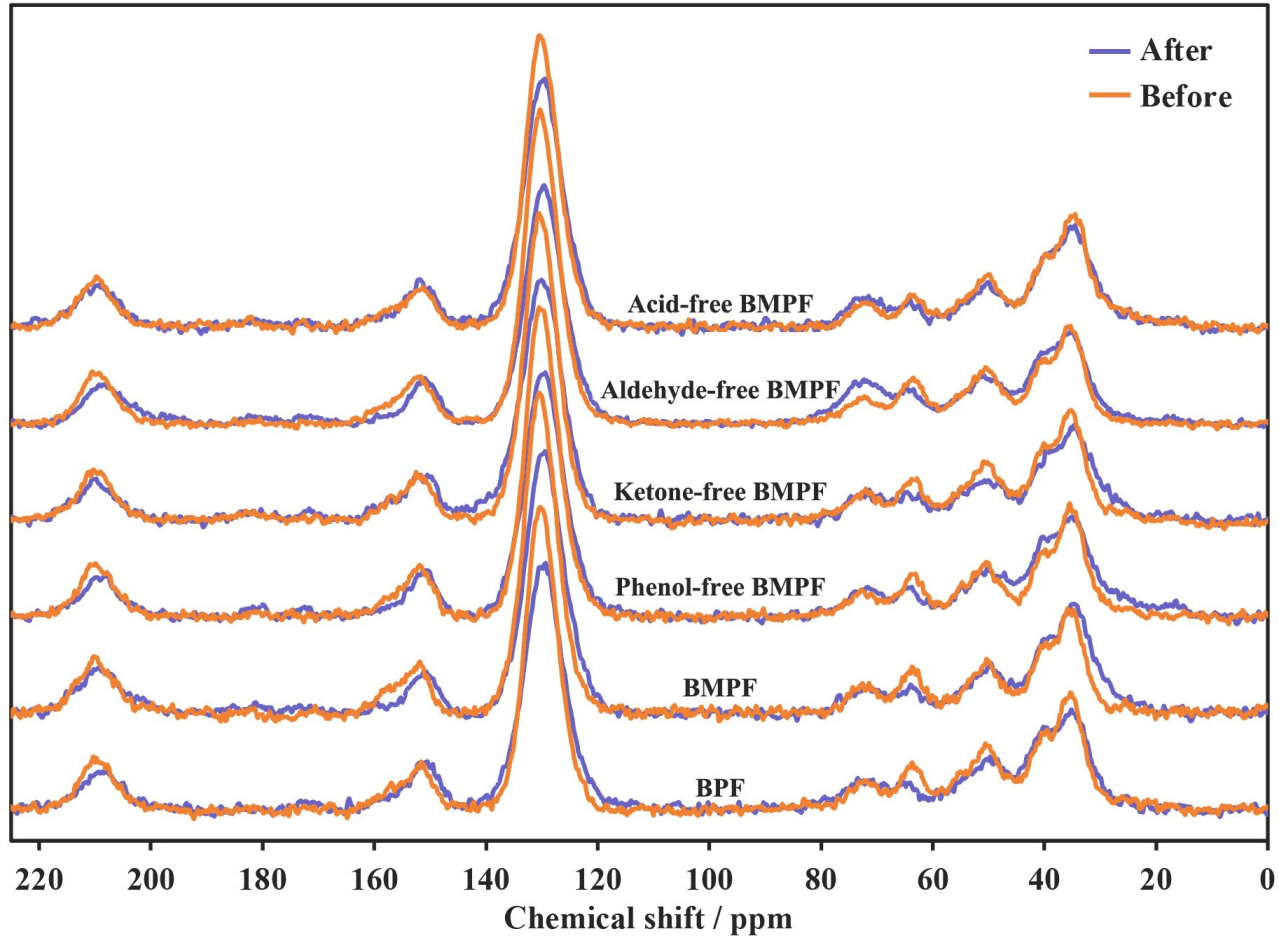
^13^C NMR images of BMPFs before and after aging for 960 h.

^13^C NMR images of BMPFs before and after aging for 960 h are displayed in Fig 2. The chemical structure changes of BMPFs are shown in Table 5 [28, 29]. The carbon spectra of BMPF and BPF were basically similar, indicating that the preparation of bio-oil model compound was reasonable, and the conclusions were the same as that of FT-IR analysis. Compared with BPF, the values of C = O (220–200 ppm and186-168 ppm) of BMPF were small, because the bio-oil contained more kinds of C = O structural substances than bio-oil model compound. Besides, the values of substituted *ortho* and *para* aromatic carbons (139–120 ppm) and methylene bridges (45–27 ppm) of BMPF were larger than those of BPF, which meant that the other components of bio-oil would still affect the resin synthesis process. However, the value of dimethylene ether bridges (77–68 ppm) increased, which might be due to the presence of ethers in the bio-oil. Compared with BMPF, the values of methylene bridges of phenol-free BMPF and aldehyde-free BMPF decreased, suggesting that the phenols and the ketones of bio-oil played a positive role in the synthesis reaction; While the values of methylene bridges of aldehyde-free BMPF and acid-free BMPF increased, indicating that the aldehydes and the acids of bio-oil had opposite effects.

**Table 5:** ^13^C NMR assignment and quantitative analysis of chemical groups for BMPF before and after aging for 960 h.

Chemical group structure	Chemical shirt (ppm)	Calculation												
		Before aging							After aging					
		0^a^	1	2	3	4	5		0	1	2	3	4	5
C = O from aldehydes/ketones	220–200	1.16	1.11	1.09	0.79	0.91	1.05		0.95	0.97	0.82	0.65	0.88	0.94
C = O from carboxylic acids	186–176	0.06	0.05	0.06	0.06	0.06	0.04		0.08	0.09	0.10	0.11	0.08	0.05
C = O from esters	176–168	0.04	0.02	0.03	0.03	0.02	0.01		0.09	0.04	0.08	0.09	0.12	0.08
Phenolic carbon	164–145	1.00	1.00	1.00	1.00	1.00	1.00		1.00	1.00	1.00	1.00	1.00	1.00
Substituted *ortho* and *para* aromatic carbons	139–120	4.72	5.43	4.52	4.20	5.32	5.60		4.12	4.52	4.24	4.03	4.69	5.04
Dimethylene ether bridges	77–68	0.50	0.46	0.22	0.45	0.39	0.43		0.49	0.41	0.42	0.34	0.37	0.36
Methylol groups	68–60	0.61	0.58	0.43	0.53	0.60	0.53		0.57	0.38	0.39	0.30	0.26	0.26
Benzylamines	60–45	1.19	1.05	1.01	0.90	1.04	1.08		1.12	0.92	0.92	0.71	0.93	0.73
Methylene bridges	45–27	2.55	2.63	2.44	2.47	2.65	2.87		2.43	2.49	2.32	2.30	2.51	2.72

^a^ 0: BPF; 1: BMPF; 2: Phenol-free BMPF; 3: Ketone-free BMPF; 4: Aldehyde-free BMPF; 5: Acid-free BMPF

After aging for 960 h, the intensity of C = O from carboxylic acids and esters (186–168 ppm) increased, which meant that some oxidative aging reaction happened during the aging of BMPFs. The intensity of C = O from aldehydes (220–200 ppm) decreased, suggesting that some aldehyde groups were converted to carboxyl or ester groups. The reduction of the substituted *ortho* and *para* aromatic carbons meant that the resin network structure was broken after aging, and small molecular substances were formed. The decreased intensity of methylene bridges indicated that the molecular chain of resin was broken after aging. Compared with BMPF, the reduction of methylene bridge of BPF was lower, indicating that the improvement on aging resistance was the result of the overall interaction of bio-oil. The reductions of methylene bridge of phenol-free BMPF, ketone-free BMPF, aldehyde-free BMPF and acid-free BMPF were higher than that of BMPF, which also confirmed this inference.

### Characteristics

The characteristics of BMPFs are shown in Table 6. The pH value of BMPFs were all increased in comparison with BPF, indicating that the acidity of bio-oil model compound was lower than that of bio-oil. The acid-free BMPF had the highest pH value because of the lack of acid compounds. Compare with BPF, the viscosity of BMPFs decreased, which might be due to the less reactions happened because of the fewer component types in the bio-oil model compounds than that in the bio-oil. Besides, the fewer long-chain substances of bio-oil model compounds also weakened the drag effect. The lower viscosity of phenol-free BMPF was due to the lack of phenolic substances, which reduced the crosslinking degree of resin. However, the ketone-free BMPF had the lowest viscosity because the ketones improved the resin fluidity as a good solvent.

**Table 6:** Characteristics of BMPFs.

Resins	Performance			
	pH (25°C)	Viscosity (25°C, mPa·s)	Solid content (%)	Formaldehyde emission (mg/L)
BPF	11.10±0.13	322±54	45.93±0.22	0.272±0.021
BMPF	11.28±0.20	136±33	46.38±0.07	0.387±0.018
Phenol-free BMPF	11.33±0.07	101±12	43.40±0.10	0.594±0.024
Ketone-free BMPF	11.32±0.03	98±18	44.80±0.12	0.452±0.032
Aldehyde-free BMPF	11.32±0.05	112±25	46.16±0.13	0.539±0.039
Acid-free BMPF	11.40±0.04	139±34	46.68±0.30	0.438±0.028

As shown in Table 6, the solid content of BMPF was higher than that of BPF, indicating that the bio-oil had a greater negative effect in comparison with the bio-oil model compounds. The phenol-free BMPF had the smallest solid content, because the lack of phenols led to the lower polymerization degree. The solid content of ketone-free BMPF was smaller than that of BMPF, which meant that the ketones had a positive effect on the resin polyreaction. Compare with BMPF, the increase in solid content of aldehyde-free BMPF and acid-free BMPF indicated that the aldehydes and the acids of bio-oil had a negative effect. The acids of bio-oil cloud fall the pH value of system, leading to the decreasing number of multi-substituted hydroxymethyl phenol in the resin addition process, and further reducing the resin crosslinking degree. The active site of formaldehyde is 2, while the reaction activity of other aldehydes in the bio-oil was lower than that of formaldehyde. This meant that the other aldehydes would become the terminator of reaction, thus reducing the resin solid content.

It can be seen from Table 6 that the formaldehyde emission of BMPF was higher than that of BPF, indicating that many substances in the bio-oil were conducive to reduce the formaldehyde emission. In other words, there were many substances in the bio-oil that could react with or adsorb formaldehyde. Besides, the formaldehyde emissions of BMPFs were all higher than that of BPF, which meant that the different components of bio-oil played differentiated positive effect on reducing the formaldehyde emission. Such as the phenols can polymerized with formaldehyde, the acids can be esterified with formaldehyde, and the ketones can promote the reaction of other substances with formaldehyde.

### Bonding strength

**Fig 3:**
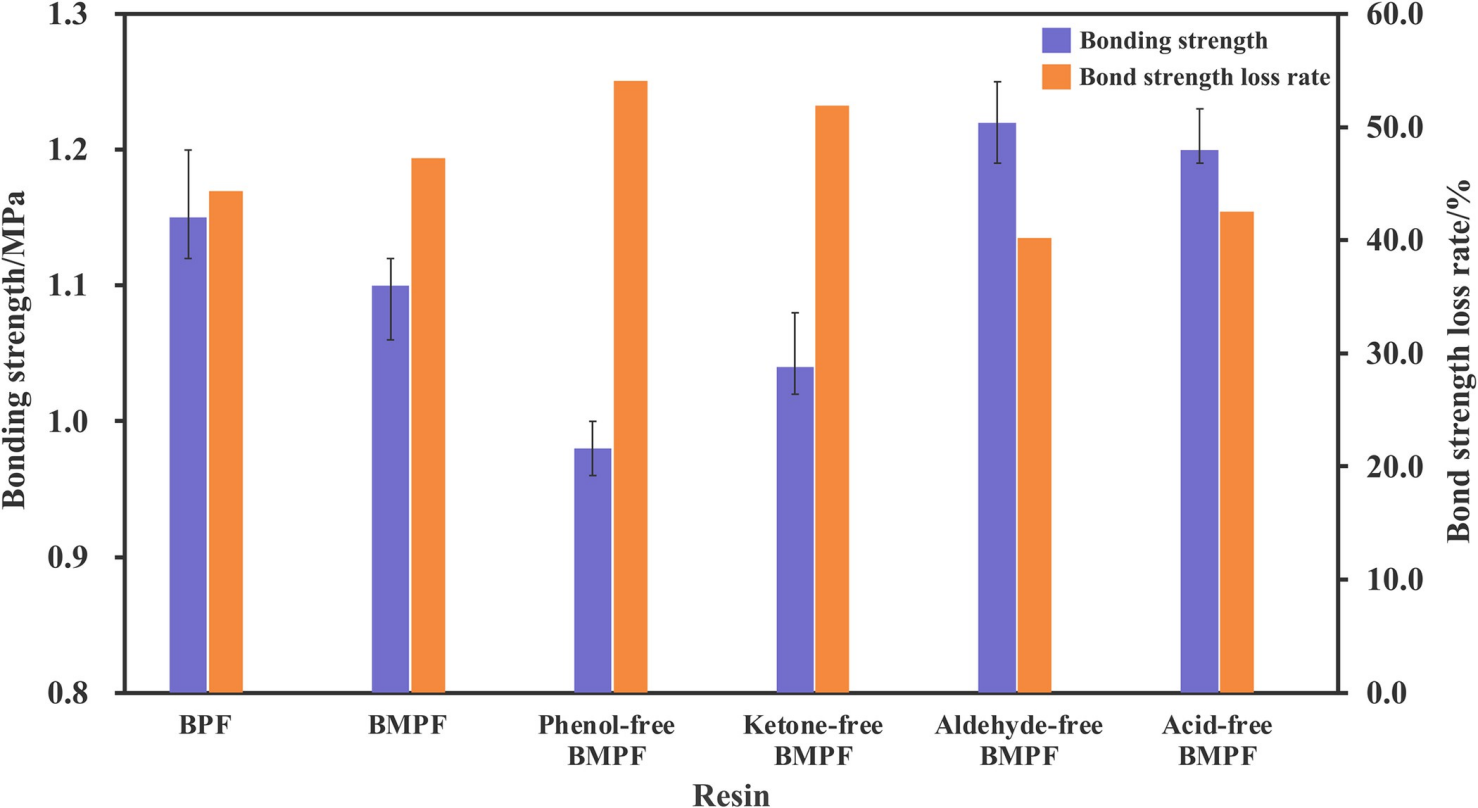
Bonding strength and its loss rate of BMPF plywood.

The bonding strength and its loss rate of BMPF plywood are shown in Fig 3. The bonding strength of BMPF plywood was lower than that of BPF plywood, indicating that the bio-oil had less influence on the resin system compare to the bio-oil model compounds. The phenol-free BMPF plywood had the lowest bonding strength because the total amount of phenolic substances of phenol-free bio-oil model was the lowest, resulting in the decrease of resin polymerization degree. Compared with BMPF plywood, the ketone-free BMPF plywood had a lower bonding strength, which suggested that the ketones could indeed promote the resin reaction. The increased bonding strength of aldehyde-free BMPF plywood meant that the aldehydes in the bio-oil had a negative effect on the bonding strength. This possible reason was that the aldehydes in the bio-oil would compete the reactive sites with formaldehyde, however, lots of the aldehydes in the bio-oil have only one active site, which blocked the further increase of resin molecular chain [30]. Compared with BMPF1 plywood, the bonding strength of acid-free BMPF plywood increased, which was due to the decrease of pH value [31].

It can be seen from Fig 3 that after 960 h of aging, the loss rate of bonding strength of BMPF plywood was higher than that of BPF plywood, suggesting that except the bio-oil model compounds, the other substances in the bio-oil also played a positive role in aging resistance. The bonding strength loss rate of phenol-free bio-oil model plywood was the biggest, which was due to the lack of phenolic content. Compared with BMPF plywood, the bonding strength loss rate of ketone-free BMPF plywood was higher because of the long-chain flexible groups in the ketones. As all known, the long-chain flexible groups could effectively improve the toughness of resin. Besides, the increased toughness could reduce the resin layer fractures caused by the stress and water-corrosion during aging, thereby improving the aging resistance of resin [32]. The decrease of bonding strength loss rate of aldehyde-free BMPF and acid-free BMPF plywood was due to the negative effect from the aldehydes and pH value on the resin synthesis reaction. However, the bonding strength loss rate of acid-free BMPF plywood was higher than that of aldehyde-free BMPF plywood, because the lower pH value could reduce the resin solubility, which slightly improved the water resistance [30].

### SEM analysis

**Fig 4:**
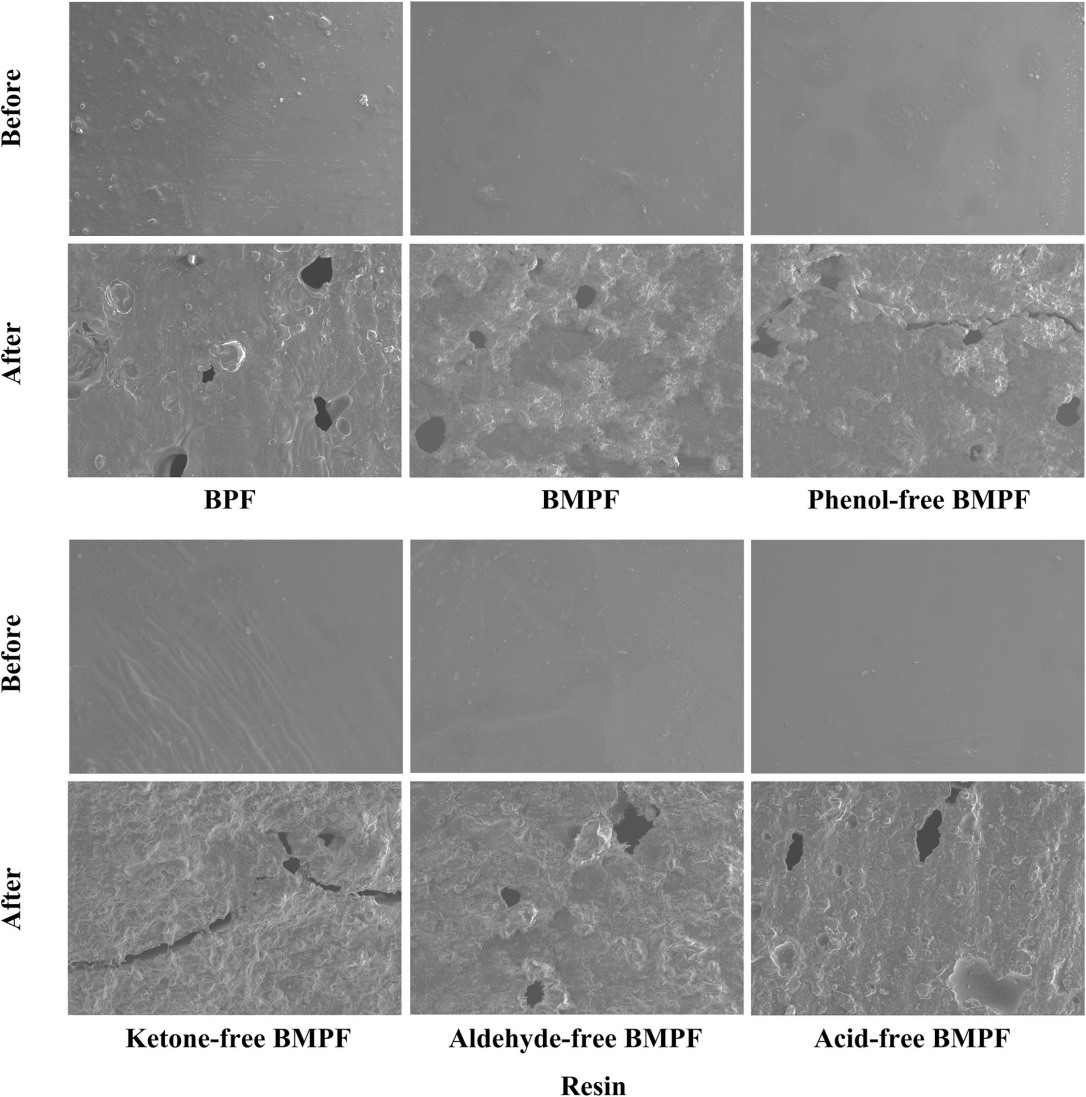
SEM images of BMPF films before and after aging for 960 h (× 500).

The apparent morphology changes of BMPF films before and after aging are shown in Fig 4. The BMPF films before aging had a smooth surface, and the degree of smoothness was higher than that of BPF film. The possible reason was that there were some solid particles in the bio-oil, which would reduce the smoothness of film surface. Besides, it also showed that the selection of bio-oil model compound was reasonable, which produced better synthesis reaction and formed a smooth surface after curing. Compared with BMPFs, the smoothness of ketone-free BMPF film surface was the lowest because the ketones could act as a good solvent and promote the resin to form a better surface.

It can be seen from Fig 4 that the surface roughness of BMPF films increased obviously after aging for 960 h, and many defects such as holes and cracks appeared. Compared with BPF, the BMPF film had a higher aging degree. Compared with BMPFs, phenol-free BMPF and, ketone-free BMPF had a greater aging degree. The reduction of phenolic substances reduced the degree of resin polymerization, thereby weakening the aging resistance. Meanwhile, the lack of ketones reduced the oily substances in the resin system, resulting in a lower water resistance.

## Conclusions

Crude bio-oil was used as a phenol substitution to synthesize the BPF resin, and its performance and characterization were analyzed by model compound method. The bio-oil components had different influence on the performance and microstructure of BMPF. The performance of BMPFs was basically the same as that of BPF, indicating that the model compounds of bio-oil were reasonably configured. Compared with BMPF, the bonding strength of phenol-free BMPF and ketone-free BMPF plywood decreased, while aldehyde-free BMPF and acid-free BMPF plywood increased. The structural analysis showed that the CH_2_ peak in FT-IR and the methylene bridges intensity in NMR of phenol-free BMPF and ketone-free BMPF were lower than that of BMPF, while the results for aldehyde-free BMPF and acid-free BMPF were opposite. These indicated that the phenols and the ketones of bio-oil had positive effects, while the aldehydes and the acids had negative effects. After aging for 960 h, compared with BMPF, the CH_2_ peak of phenol-free BMPF, ketone-free BMPF, aldehyde-free BMPF and acid-free BMPF decreased significantly and their reductions of methylene bridge was higher, which meant that all components of bio-oil could improve the aging resistance of BMPF inordinately.

## Supporting information

S1 Data (XLSX)
